# Supplementary material for: The dynamic and structural properties of axonemal tubulins support the high length stability of cilia
Source: Nat Commun. 2019 Apr 23;10:1838. doi: 10.1038/s41467-019-09779-6 (PMC6479064; doi:10.1038/s41467-019-09779-6)
Supplement: Supplementary file 13 — Supplementary Information [file 41467_2019_9779_MOESM13_ESM.pdf]

The dynamic and structural properties of axonemal tubulins support the high length stability of cilia

Orbach et al.

Supporting Information

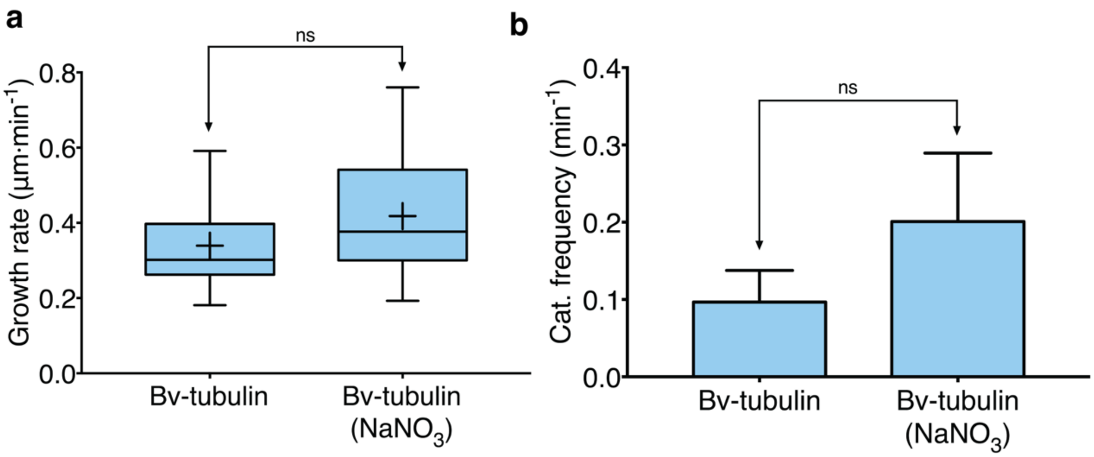

Supplementary Figure 1

Effect of $NaNO_3$ on mammalian brain tubulin. Tubulin was incubated in 500 mM $NaNO_3$ solution for 2 h, followed by size exclusion and buffer exchange. (**a**) Tukey plot showing growth rate (*n*=65, 106) and (**b**) Bar plot showing catastrophe frequency of 9 μM bovine brain tubulin with or without treatment with $NaNO_3$. Means (marked as "+") were derived from three independent experiments, middle line represents the median. Error bars represent S.D. replicates.

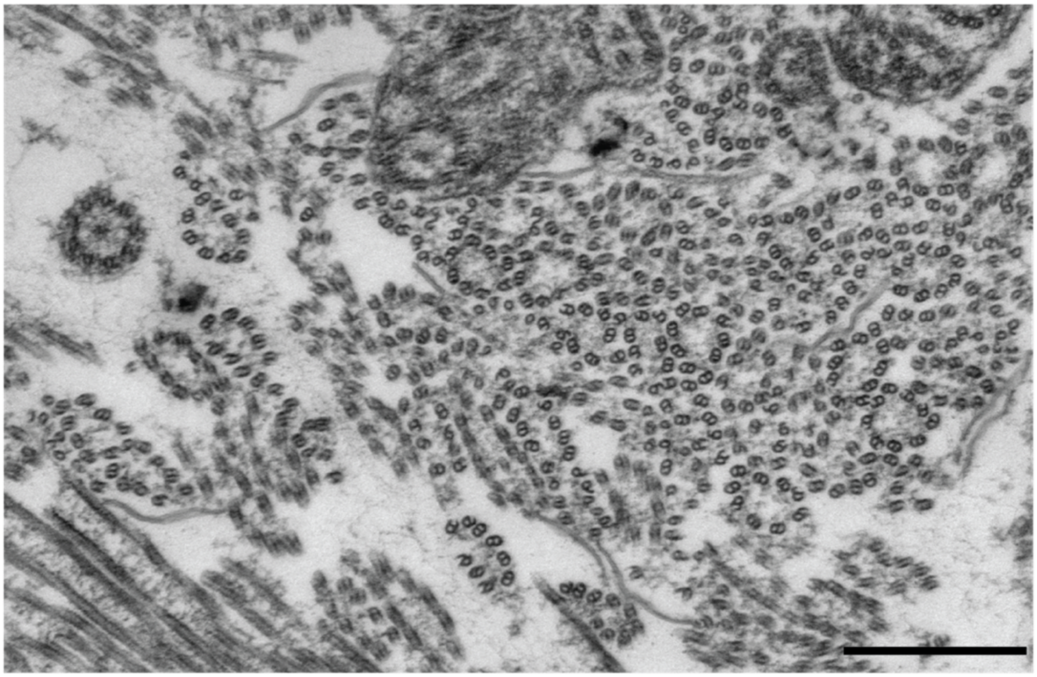

Supplementary Figure 2

Differential extraction of axonemal tubulin. TEM micrograph of axonemes treated with 330 mM $NaNO_3$. scale bar: 500 nm.

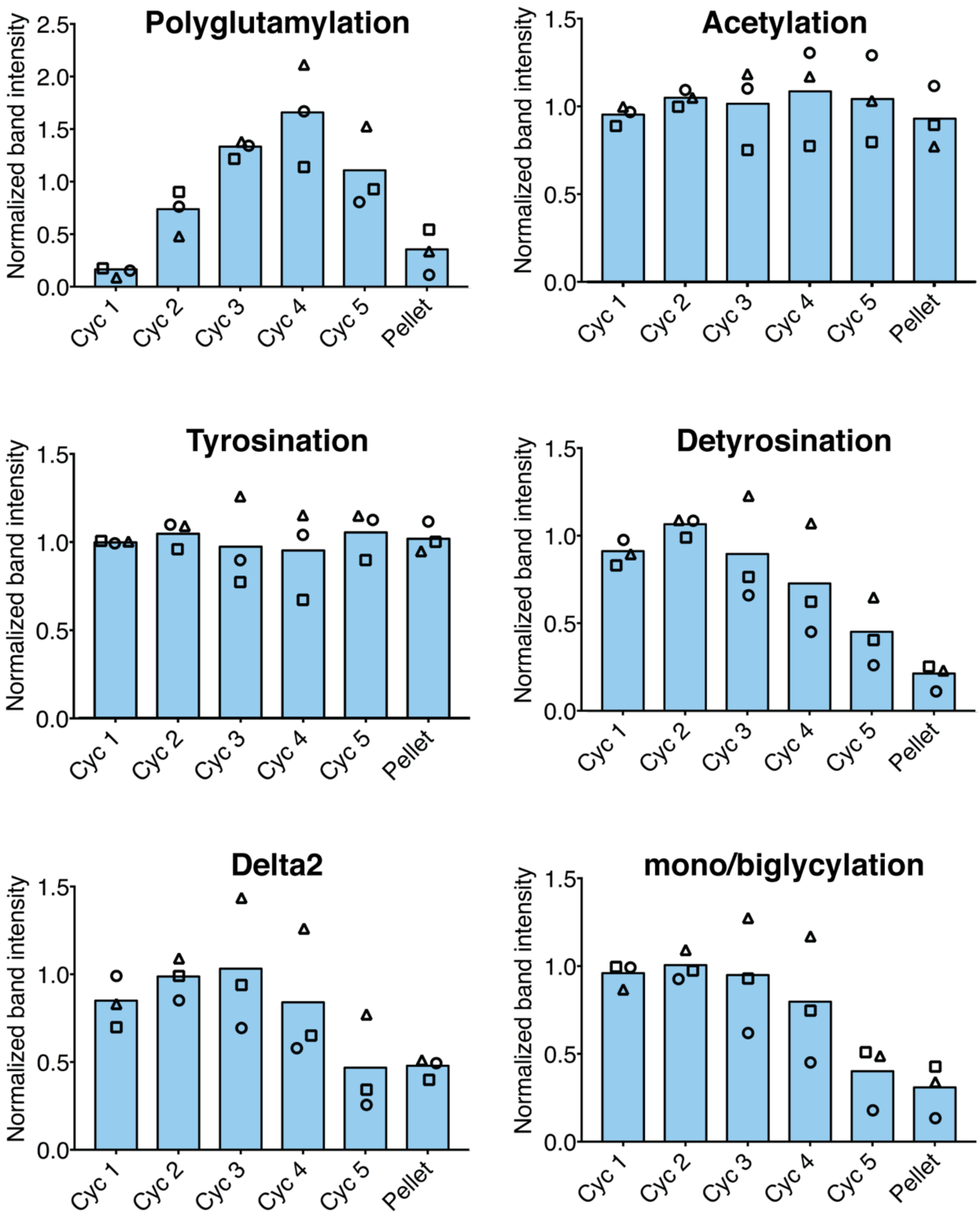

Supplementary Figure 3

Distribution of post-translational modifications between fractions. Average normalized band intensity of immunoblots. Band intensities were normalized against each other, and then against the α-tubulin band intensity. Means were derived from three independent purifications (different symbol for each purification).

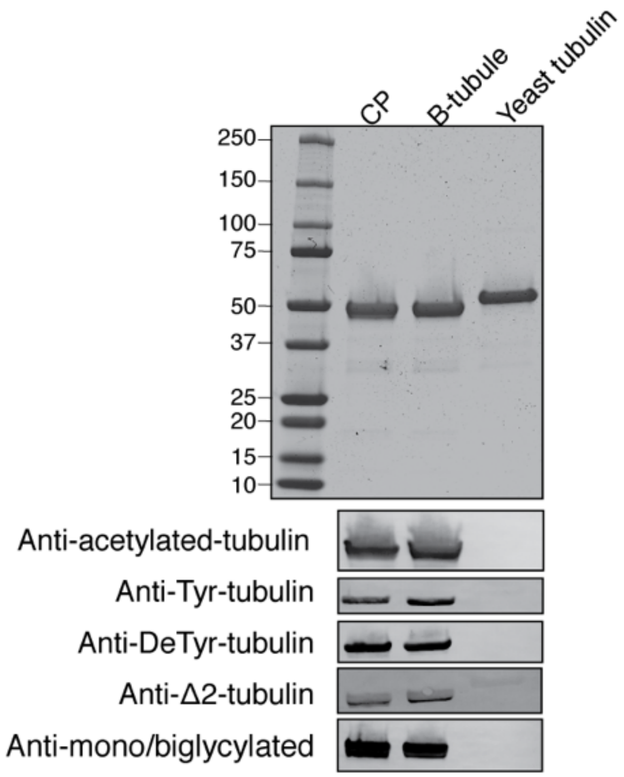

Supplementary Figure 4

Specificity of antibodies to various PTMs. SDS-PAGE and immunoblots of tubulins purified from the CP, B-tubule and yeast. Yeast tubulin has no PTMs and provide a background control. Additionally, previous study from our lab showed the specificity of these antibodies following enzymatic treatment with SIRT2 for deacetylation, and by removing the C-terminal tail (CTT), which is subjected to the other PTMs, using subtilisin[1].

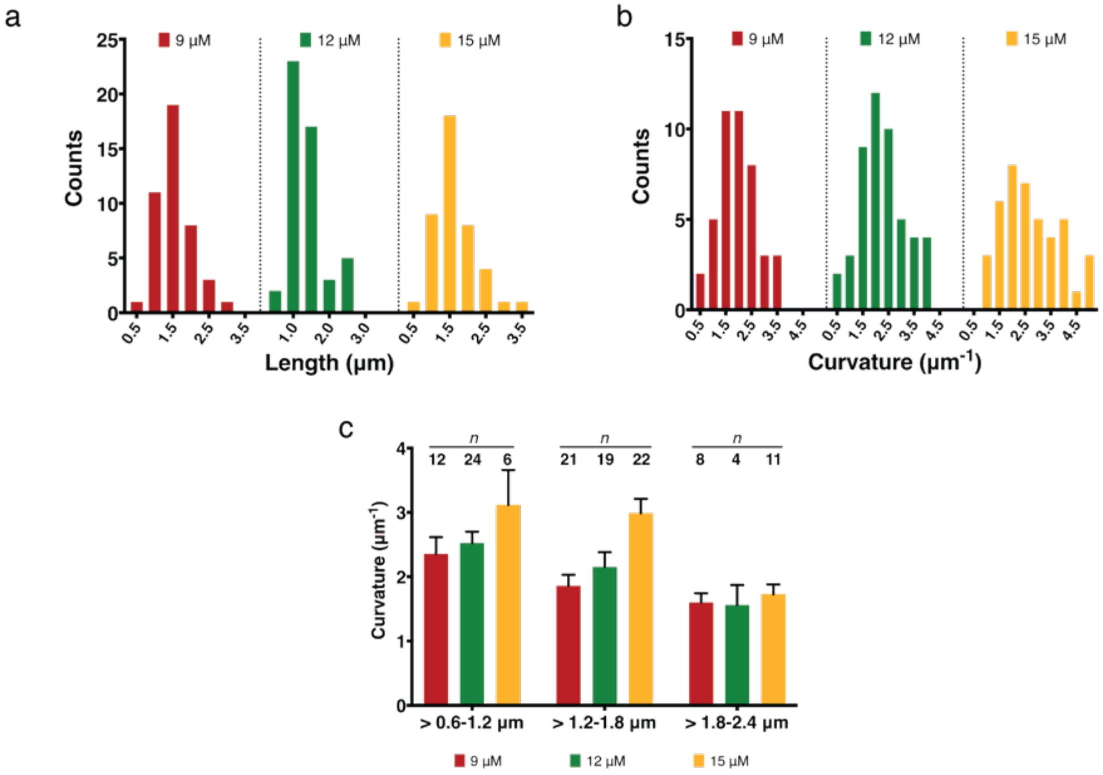

Supplementary Figure 5

Growth of curved PFs polymerized with axonemal tubulins. (**a**) Histogram showing distributions of lengths of curved regions for different tubulin concentrations. (**b**) Histogram showing distributions of curvature for different tubulin concentrations. (**c**) Mean curvature for each tubulin concentration and for different tip lengths show linear trend.

Supplementary Table 1. Mass spectrometry analysis of axonemal tubulin from the central pair

Accession	Description	Score	Coverage %	# Unique Peptides	Total Peptides	Protein Length	MW [kDa]
Q540H1	Tubulin alpha chain OS=Chlamydomonas reinhardtii OX=3055 GN=TUA1 PE=3 SV=1	90513	82	33	1892	451	49.6
A8IXZ0	Tubulin beta chain OS=Chlamydomonas reinhardtii OX=3055 GN=TUB1 PE=3 SV=1	79389	84	37	2583	443	49.6
NP_463397.1	PTS system mannose-specific transporter subunit IID [Salmonella enterica subsp. enterica serovar Typhimurium str. LT2]	91	3	1	5	278	30.9
P02769	Serum albumin OS=Bos taurus GN=ALB PE=1 SV=4	34	2	1	1	607	69.2
NP_461896.1	L-fuculokinase [Salmonella enterica subsp. enterica serovar Typhimurium str. LT2]	32	2	1	2	472	51.6
809009895	membrane protein [Erwinia tracheiphila]	24	2	1	5	338	35.8
NP_462668.2	PTS system mannose-specific transporter subunit IID [Salmonella enterica subsp. enterica serovar Typhimurium str. LT2]	22	4	1	2	285	30.7
NP_462444.1	gluconate operon transcriptional repressor [Salmonella enterica subsp. enterica serovar Typhimurium str. LT2]	20	2	1	3	331	36.5

Supplementary Table 2. Mass spectrometry analysis of axonemal tubulin from the B-tubule

Accession	Description	Score	Coverage %	# Unique Peptides	Total Peptides	Protein Length	MW [kDa]
Q540H1	Tubulin alpha chain OS=Chlamydomonas reinhardtii OX=3055 GN=TUA1 PE=3 SV=1	108024	82	32	2264	451	49.6
A8IXZ0	Tubulin beta chain OS=Chlamydomonas reinhardtii OX=3055 GN=TUB1 PE=3 SV=1	103333	84	36	3148	443	49.6
136429	RecName: Full=Trypsin; Flags: Precursor	117	9	1	2	231	24.4
P02769	Serum albumin OS=Bos taurus GN=ALB PE=1 SV=4	113	5	2	2	607	69.2
NP_463397.1	PTS system mannose-specific transporter subunit IID [Salmonella enterica subsp. enterica serovar Typhimurium str. LT2]	67	3	1	4	278	30.9
NP_490570.1	conjugal transfer protein TraV [Salmonella enterica subsp. enterica serovar Typhimurium str. LT2]	63	4	1	2	171	18.4
NP_461873.1	enolase [Salmonella enterica subsp. enterica serovar Typhimurium str. LT2]	37	11	2	29	432	45.6
NP_461896.1	L-fuculokinase [Salmonella enterica subsp. enterica serovar Typhimurium str. LT2]	29	2	1	3	472	51.6
NP_459546.1	diguanylate cyclase/phosphodiesterase domain-containing protein [Salmonella enterica subsp. enterica serovar Typhimurium str. LT2]	26	8	1	1	103	11.6
NP_462444.1	gluconate operon transcriptional repressor [Salmonella enterica subsp. enterica serovar Typhimurium str. LT2]	24	2	1	2	331	36.5
NP_462668.2	PTS system mannose-specific transporter subunit IID [Salmonella enterica subsp. enterica serovar Typhimurium str. LT2]	22	4	1	2	285	30.7
NP_459936.1	DNA translocase FtsK [Salmonella enterica subsp. enterica serovar Typhimurium str. LT2]	21	1	1	1	1351	148.2

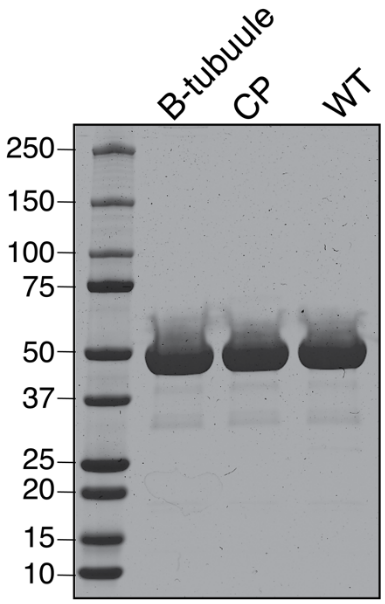

Supplementary Figure 6

SDS-PAGE of axonemal tubulin. SDS-PAGE of high tubulin load (15 µg) shows only low molecular weight bands, which are associated with tubulin degradation.

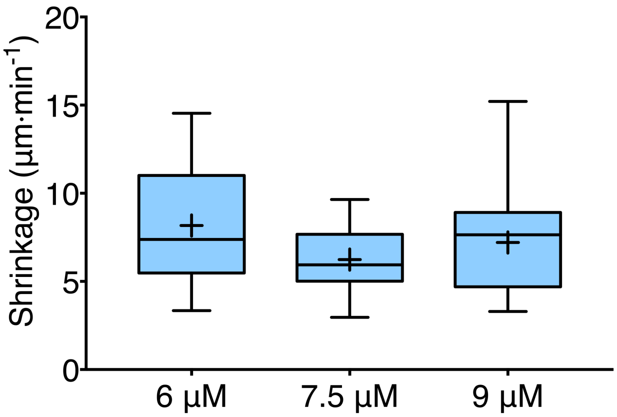

Supplementary Figure 7

Shrinkage rate of axonemal MTs. Tukey plot showing shrinkage rate of axonemal MTs in different tubulin concentrations. Means (marked as "+") derived from three independent technical replicates (*n*=34, 24, 24), middle lines represent the median.

a

b

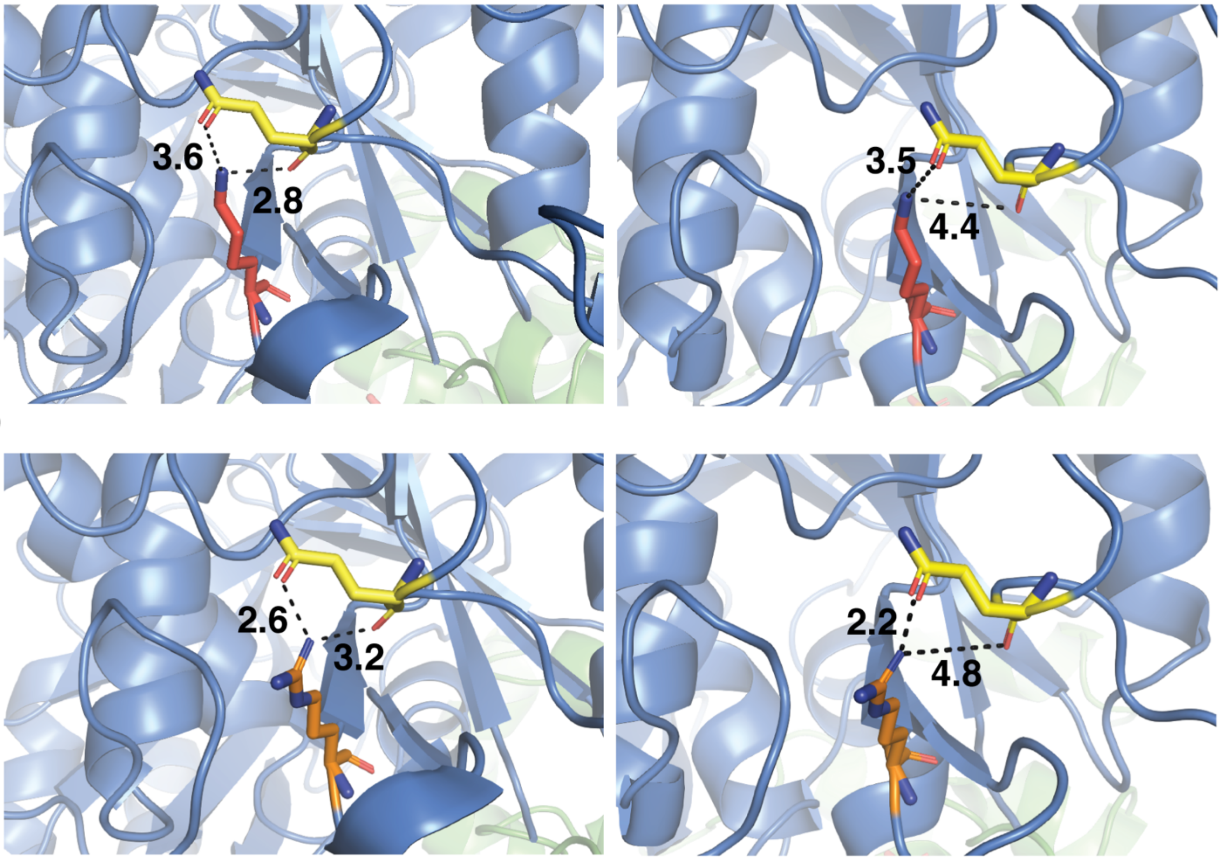

Supplementary Figure 8

Substitution of Lys-58 with Arg residue. (**a**) Lys-58 (red) in the H1'-S2 loop interacts with Gln-280 (yellow) and stabilizes the lateral contacts between neighboring subunits at the GDP-Pi state (PDB: 6EVX) (left), Whereas in the GDP state this interaction is no longer involved due to the long distance (PDB: 6EVZ) (right) [2]. (**b**) Substitution of Lys-58 to Arg (orange) might lead to more stable interactions with Gln-280 in the GDP-Pi state due to the short distance (left) or even in the GDP state (right). Each substitution was plotted on the solved protein structure for the GDP•Pi-DCX-MT (PDB: 6EVX) using PyMOL software (1.1r1, http://www.pymol.org/).

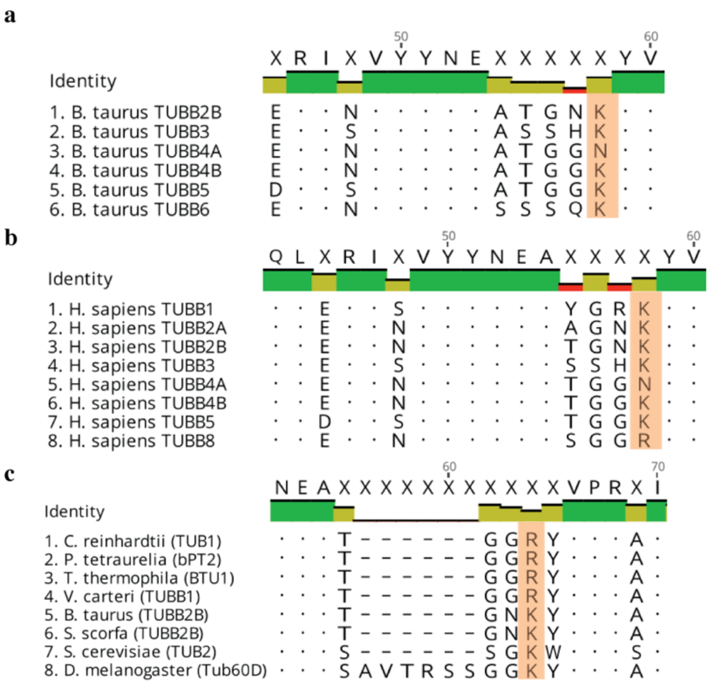

Supplementary Figure 9

Tubulin sequence alignment for Lys-58. (**a**) Bovine brain tubulin isotypes include mainly lysine (K) residue at position 58 of β-tubulin, except TUBB4A that includes Asp (N) (**b**) Human tubulin isotypes include mainly lysine residue at position 58 of β-tubulin, except for TUBB4A that includes Asp (N), and TUBB8 that includes Arg (R). (**c**) Ciliates from different phylogenetic background have Arg-58 for all tubulin isotypes, while other organisms have mainly Lys-58 and sometimes Asp (but no Arg) for the different tubulin isotypes.

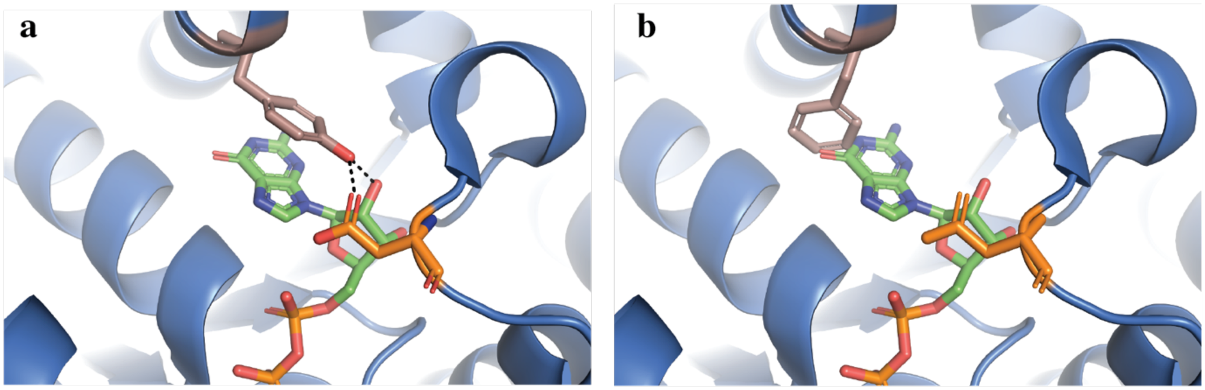

Supplementary Figure 10

Substitution of Tyr-222 with Phe residue. (**a**) Tyr-222 (salmon) in helix H7 at the E-site interacts with the nucleobase and Asp-179 (orange) [3]. (**b**) Substitution of Tyr-222 to Phe-222 (salmon) might change the orientation of GTP in the E-site, and consequently to affect the hydrolysis process of GTP to GDP (green). Due to the missing interactions of Phe-222, Asp-179 is more available to longitudinal interactions.
Substitution was plotted on the solved protein structure for the GDP-sT_2R (PDB: 3RYI) using PyMOL software (http://www.pymol.org/).

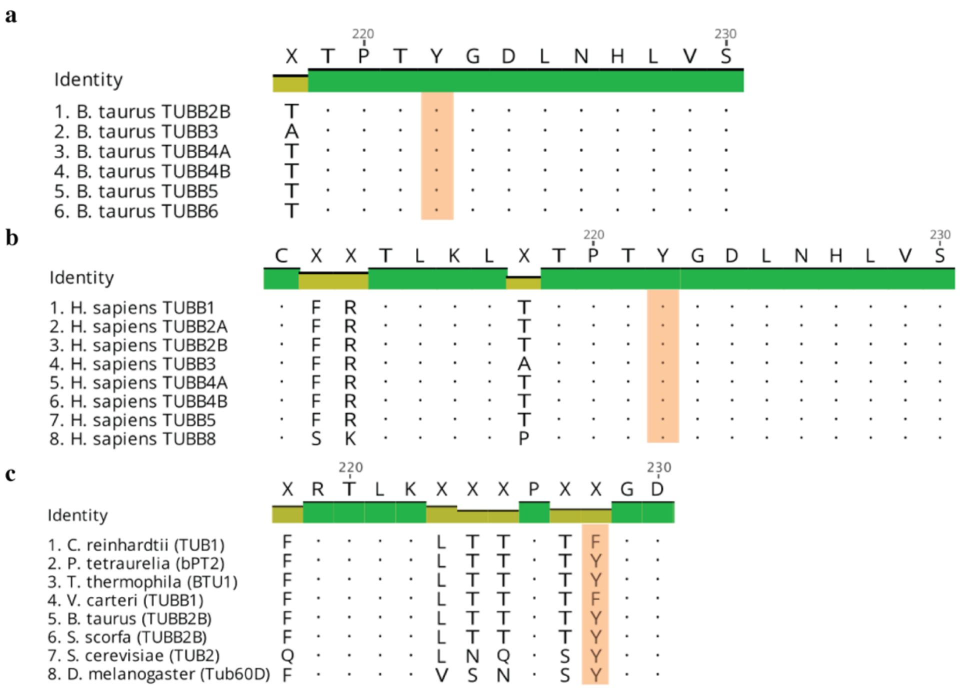

Supplementary Figure 11

Tubulin sequence alignment for Tyr222. Bovine (**a**) and human (**b**) tubulin isotypes include Tyr (Y) residue at position 222 of β-tubulin. (**c**) Only *Chlamydomonas reinhardtii* and *Volvox carteri* include Phe (F) residue at position 222, while other studied organisms have Tyr-222 in all tubulin isotypes.

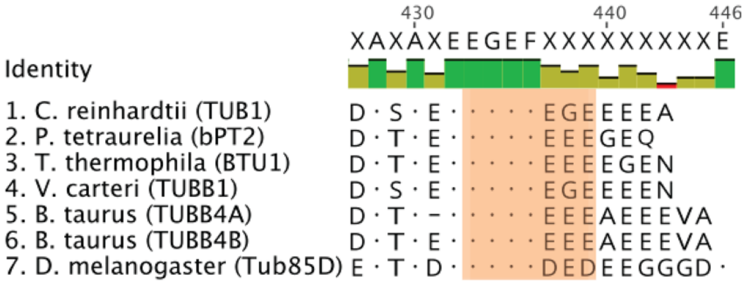

Supplementary Figure 12

Tubulin sequence alignment for 'EGEFXXX' motif. All organisms have 'EGEFX(X/G)X' motif at the C-terminal tail of β-tubulin of axonemal tubulins.

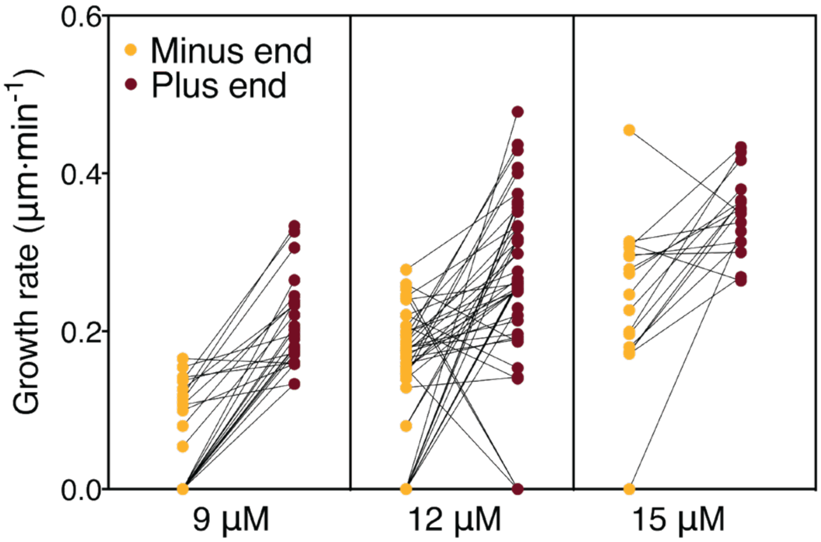

Supplementary Figure 13

Identification of plus and minus ends. Dual-labeled GMPCPP stabilized bovine brain MTs seeds were used to follow the growth rate at minus end (blue) and the plus end (red) of axonemal tubulin at different tubulin concentrations.

References

1. Alper, J. D., Decker, F., Agana, B. & Howard, J. The motility of axonemal dynein is regulated by the tubulin code. *Biophys. J.* **107,** 2872–2880 (2014).
2. Manka, S. W. & Moores, C. A. The role of tubulin-tubulin lattice contacts in the mechanism of microtubule dynamic instability. *Nat. Struct. Mol. Biol.* **114,** 977 (2018).
3. Nawrotek, A., Knossow, M. & Gigant, B. The determinants that govern microtubule assembly from the atomic structure of GTP-tubulin. *J. Mol. Biol.* **412,** 35–42 (2011).
