# Supplementary material for: The dynamic and structural properties of axonemal tubulins support the high length stability of cilia
Source: Nat Commun. 2019 Apr 23;10:1838. doi: 10.1038/s41467-019-09779-6 (PMC6479064; doi:10.1038/s41467-019-09779-6)
Supplement: Supplementary file 14 — Description of Additional Supplementary Files [file 41467_2019_9779_MOESM14_ESM.docx]

**Title:** Supplementary Movie 1:
**Description:** Curved tip structure of a growing axonemal microtubule.

**Title:** Supplementary Movie 2:
**Description:** Dynamic bovine microtubules.

**Title:** Supplementary Movie 3:
**Description:** Fast growth phases of an axonemal microtubule.

**Title:** Supplementary Movie 4:
**Description:** Fast growth phase after catastrophe event of an axonemal microtubule.

**Title:** Supplementary Movie 5:
**Description:** Fast growth of protofilaments along polymerized axonemal protofilaments.

**Title:** Supplementary Movie 6:
**Description:** Stable protofilaments following catastrophe event of an axonemal microtubule.

**Title:** Supplementary Movie 7:
**Description:** Stable curved protofilaments following catastrophe event of an axonemal microtubule (fast imaging).

**Title:** Supplementary Movie 8:
**Description:** Stable protofilaments following catastrophe event of an axonemal microtubule (fast imaging).

**Title:** Supplementary Movie 9:
**Description:** Curved tip structure of a growing axonemal microtubule using TIRF microscopy.

**Title:** Supplementary Movie 10:
**Description:** Fast growth phase of an axonemal microtubule using TIRF microscopy.

**Title:** Supplementary Movie 11:
**Description:** Curved tip structure of a growing axonemal microtubule using cycled tubulin.

**Title:** Supplementary Movie 12:
**Description:** Fast growth phase of an axonemal microtubule using cycled tubulin.
