# Supplementary material for: The dynamic and structural properties of axonemal tubulins support the high length stability of cilia
Source: Nat Commun. 2019 Apr 23;10:1838. doi: 10.1038/s41467-019-09779-6 (PMC6479064; doi:10.1038/s41467-019-09779-6)
Supplement: Supplementary file 15 — Reporting Summary [file 41467_2019_9779_MOESM15_ESM.pdf]

nature research

Corresponding author(s): Jonathon Howard

Last updated by author(s): Mar 18, 2019

Reporting Summary

Nature Research wishes to improve the reproducibility of the work that we publish. This form provides structure for consistency and transparency in reporting. For further information on Nature Research policies, see Authors & Referees and the Editorial Policy Checklist.

Statistics

For all statistical analyses, confirm that the following items are present in the figure legend, table legend, main text, or Methods section.

n/a	Confirmed	
☐	☒	The exact sample size (*n*) for each experimental group/condition, given as a discrete number and unit of measurement
☐	☒	A statement on whether measurements were taken from distinct samples or whether the same sample was measured repeatedly
☐	☒	The statistical test(s) used AND whether they are one- or two-sided<br>*Only common tests should be described solely by name; describe more complex techniques in the Methods section.*
☒	☐	A description of all covariates tested
☒	☐	A description of any assumptions or corrections, such as tests of normality and adjustment for multiple comparisons
☐	☒	A full description of the statistical parameters including central tendency (e.g. means) or other basic estimates (e.g. regression coefficient) AND variation (e.g. standard deviation) or associated estimates of uncertainty (e.g. confidence intervals)
☒	☐	For null hypothesis testing, the test statistic (e.g. *F*, *t*, *r*) with confidence intervals, effect sizes, degrees of freedom and *P* value noted<br>*Give P values as exact values whenever suitable.*
☒	☐	For Bayesian analysis, information on the choice of priors and Markov chain Monte Carlo settings
☒	☐	For hierarchical and complex designs, identification of the appropriate level for tests and full reporting of outcomes
☒	☐	Estimates of effect sizes (e.g. Cohen's *d*, Pearson's *r*), indicating how they were calculated

Our web collection on statistics for biologists contains articles on many of the points above.

Software and code

Policy information about availability of computer code

Data collection	Microscopy imaging was powered by NIS-Elements Confocal software, v.4.30 (Nikon, Japan).
Data analysis	MSD analysis was done using msd analyzer package; The end position of the microtubules was tracked FIESTA; mass-spec analysis was done using MASCOT; Sequence alignments were made in Geneious 9.1.8; All statistical tests were made in Prism7 (GraphPad Software, La Jolla, CA, USA). All images were processed using Fiji.

For manuscripts utilizing custom algorithms or software that are central to the research but not yet described in published literature, software must be made available to editors/reviewers. We strongly encourage code deposition in a community repository (e.g. GitHub). See the Nature Research guidelines for submitting code & software for further information.

Data

Policy information about availability of data

All manuscripts must include a data availability statement. This statement should provide the following information, where applicable:

- Accession codes, unique identifiers, or web links for publicly available datasets
- A list of figures that have associated raw data
- A description of any restrictions on data availability

The data that support the findings of this study are available from the corresponding author upon reasonable request.

Field-specific reporting

Please select the one below that is the best fit for your research. If you are not sure, read the appropriate sections before making your selection.

☒ Life sciences ☐ Behavioural & social sciences ☐ Ecological, evolutionary & environmental sciences

For a reference copy of the document with all sections, see nature.com/documents/nr-reporting-summary-flat.pdf

Life sciences study design

All studies must disclose on these points even when the disclosure is negative.

Sample size	The sample size was chosen based on common practice in the field
Data exclusions	No Data was excluded
Replication	To ensure reproducibility, the experiments were performed over several months. All replication attempts were successful
Randomization	Randomization was not relevant for this study, as samples were never needed to be distributed into experimental groups.
Blinding	Blinding was ensured by randomization of choice of the region of the interest (ROI), within each ROI the entire MTs population was analyzed

Reporting for specific materials, systems and methods

We require information from authors about some types of materials, experimental systems and methods used in many studies. Here, indicate whether each material, system or method listed is relevant to your study. If you are not sure if a list item applies to your research, read the appropriate section before selecting a response.

Materials & experimental systems

n/a	Involved in the study	
☐	☒	Antibodies
☒	☐	Eukaryotic cell lines
☒	☐	Palaeontology
☒	☐	Animals and other organisms
☒	☐	Human research participants
☒	☐	Clinical data

Methods

n/a	Involved in the study	
☒	☐	ChIP-seq
☒	☐	Flow cytometry
☒	☐	MRI-based neuroimaging

Antibodies

Antibodies used	anti-acetylated-tubulin (clone 6-11B-1; MilliporeSigma); anti-polyglutamylation (clone GT335;AdipoGen); anti-detyrosinated tubulin(MilliporeSigma); Gly-Pep1 anti-mono/biglcylated tubulin (AdipoGen); anti-delta2 tubulin (MilliporeSigma); anti-tyrosinated tubulin (MilliporeSigma); anti-tubulin (clone B-5-1-2;MilliporeSigma)
Validation	anti-acetylated-tubulin - commercially tested for WB and confirmed against endogenous tubulin in Chlamydomonas; anti-polyglutamylation - commercially tested for WB and no specificity to particular tubulin isoforms nor to tubulin from particular species are observed (see also: Lechtreck & Geimer Cell Motil Cytoskeleton 47:219-235 2000); anti-detyrosinated tubulin - commercially tested for WB and expected to cross-react with mammals, sea urchin and plants (Alper et al. Biophys J. 107:2872-2880); Gly-Pep1 anti-mono/biglcylated tubulin - commercially tested for WB and recognizes mono or bi-glycylated tubulins (see also: Gadadhar et al. J Cell Biol 216: 2701-2713); anti-delta2 tubulin - commercially tested for WB and confirmed against Mammals, sea urchin and plants (see also: Alper et al. Biophys J. 107:2872-2880); anti-tyrosinated tubulin - commercially tested for WB and expected to cross-react with a range of species based on sequence homology (see also: Alper et al. Biophys J. 107:2872-2880); anti-tubulin - commercially tested for IF, RIA, WB and confirmed against endogenous tubulin in Chlamydomonas;
